# Supplementary figures and images for: The role of perceived threat and self-efficacy in the use of Insecticide Treated Bednets (ITNs) to prevent malaria among pregnant women in Tororo District, Uganda
Source: PLoS One. 2023 Jul 26;18(7):e0289097. doi: 10.1371/journal.pone.0289097 (PMC10370871; doi:10.1371/journal.pone.0289097)

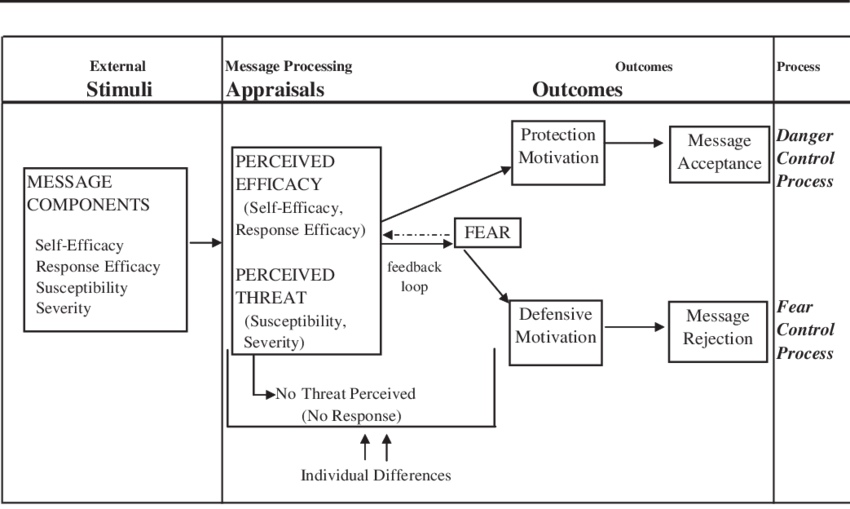

Supplement: S1 Fig — (TIF) [file pone.0289097.s002.tif]
