# Supplementary material for: The role of perceived threat and self-efficacy in the use of Insecticide Treated Bednets (ITNs) to prevent malaria among pregnant women in Tororo District, Uganda
Source: PLoS One. 2023 Jul 26;18(7):e0289097. doi: 10.1371/journal.pone.0289097 (PMC10370871; doi:10.1371/journal.pone.0289097)
Supplement: S1 Questionnaire — (DOCX) [file pone.0289097.s003.docx]

Appendix 1 - QUESTIONNAIRE

**MALARIA SBCC SURVEY**

**INDIVIDUAL QUESTIONNAIRE**

|  |  | | |  |  | |  |
| --- | --- | --- | --- | --- | --- | --- | --- |
| **INTERVIEWER VISITS** | | | | | | | |
|  | **1** | | **2** | | **3** | | **FINAL VISIT** |
|  |  | |  | |  | |  |
| DATE |  | |  | |  | | DAY \|___\|___\| |
|  |  | |  | |  | | MONTH \|___\|___\| |
|  |  | |  | |  | | YEAR \|___\|___\|___\|___\| |
|  |  | |  | |  | |  |
| INTERVIEWER’S NAME |  | |  | |  | | INT. NUMBER \|___\|___\|___\| |
|  |  | |  | |  | |  |
| RESULT* |  | |  | |  | | RESULT \|___\| |
|  |  | |  | |  | |  |
| NEXT VISIT DATE |  | |  | |  | | TOTAL NUMBER OF VISITS = \|___\| |
| TIME |  | |  | |  | |  |
|  |  | | |  |  | |  |
| ***RESULT CODES**  1 COMPLETED 3 REFUSED 5  2 POSTPONED 4 PARTLY COMPLETED (SPECIFY) | | | | | | | |
| **SUPERVISOR** | | **RESEARCH ASSISTANT** | | | | **KEYED BY** | |
| NAME \|___\|___\| | | \|___\|___\| | | | | \|___\|___\| | |
|  |  | | |  |  | |  |
|  |  | | |  |  | |  |
|  |  | | |  |  | |  |
|  |  | | |  |  | |  |
|  |  | | |  |  | |  |
|  |  | | |  |  | |  |
|  |  | | |  |  | |  |
|  |  | | |  |  | |  |
|  |  | | |  |  | |  |

|  |  |  |  |  |
| --- | --- | --- | --- | --- |
| **SECTION 1: RESPONDENT’S BACKGROUND** | | | | |

| **NO.** | **QUESTION** | **CODING CATEGORIES** | **SKIP** |
| --- | --- | --- | --- |
| 101 | RECORD THE TIME | HOURS \|___\|___\|  MINUTES \|___\|___\| |  |
| **Let us begin. Please remember that everything you tell me will be kept secret.** | | | |
| 102 | How old were you at your last birthday? | RECORD AGE IN COMPLETED YEARS \|___\|___\| |  |
| 103 | How many children have you given birth to? | 0 (pregnant with 1^st^ child) …………………..1  1…………………………………………….…………..2  2…………………………………………..…………….3  3………………………………………..……………….4  4…………………………………………………………5  5…………………………………………..…………….6  6………………………………………………………..7  7………………………………………………………..8  8 or more……………………………………………9 | Skip to 106 |
| 104 | How old were you when you had your first child? | 14 or Younger….……………………….……….1  15………………………………………………………2  16…………………………………………………….3  17……………………………………………………..4  18……………………………………………………5  19……………………………………………………..620 and over……………………………………….7 |  |
| 105 | What is the age of your youngest child? | RECORD AGE IN COMPLETED YEARS \|___\|___\| |  |
| 106 | Have you ever attended formal school? | YES 1  NO 2 | 🡪skip to 108 |
| 107 | What is the highest level of education that you attained? | PRIMARY INCOMPLETE 2  COMPLETED PRIMARY 3  SECONDARY INCOMPLETE 4  COMPLETED SECONDARY 5  MORE THAN SECONDARY 6  DON’T KNOW 9 |  |
| 108 | Does your household have:  a) Electricity?  b) A radio?  c) A cassette player?  d) A television?  e) A mobile phone?  f) A fixed phone?  g) A refrigerator?  h) A table?  i) A chair?  j) A sofa set?  k) A bed?  l) A cupboard?  m) A clock? | : YES/ NO  a) ELECTRICITY . . . . . . . . . . . . . . . …………….....1/ 2  b) RADIO . . . . . . . . . . . . . . . . . . . . . ……………...1/ 2  c) CASSETTE PLAYER . . . . . . . . . ……………….…..1/ 2  d) TELEVISION . . . . . . . . . . . . . . . ..………………..1/ 2  e) MOBILE PHONE . . . . . . . . . . . . . . . .……………1/ 2  f) FIXED PHONE . . . . . . . . . . . . . . . …………………1/ 2  g) REFRIGERATOR . . . . . . . . . . . . ....……………….1/ 2  h) TABLE . . . . . . . . . . . . . . . . . ………………….…...1/ 2  i) CHAIRS . . . . . . . . . . . . . . . . . . …………………..…1/ 2  j) SOFA SET . . . . . . . . . . . . . . . . ……………………...1/ 2  k) BED . . . . . . . . . . . . . . . . . . . ……………………….1/ 2  l) CUPBOARD . . . . . . . . . . . . . . . . ..………………...1/ 2  m) CLOCK…………... . . . . . . . . ………….………………1/ 2 |  |
| 109 | Are you currently married or living together with someone as if married? | yes 1  No 2  Don’t know 9 |  |
| 110 | How many people, including all the children, live in your home? | 1…………………………………………………..1  2-5………………………………………………..2  6-10………………………………………………3  10 or more……………………………………..4 |  |

| **SECTION 2: MOSQUITO NET ACCESS AND USE** |
| --- |

| **NO.** | **QUESTION** | **CODING CATEGORIES** | **SKIP** |
| --- | --- | --- | --- |
| 201 | Did you sleep under a mosquito net last night? | yes 1  No 2  Don’t know 9 | 🡪203 |
| 202 | What is the main reason that you did not sleep under a mosquito net last night?  **RECORD ONE ANSWER** | TOO HOT 1  TOO COLD 2  NOT ENOUGH NETS 3  NET NOT HUNG UP 4  NET USED BY SOMEONE ELSE 5  net WORN OUT/POOR CONDITION 6  NET BAD FOR HEALTH 7  OTHER 8  (SPECIFY)  don’t know 9 |  |
| 203 | In general, how often do you sleep under a mosquito net – every night, most nights, some nights, very few nights or never? | every night 1  most nights 2  some nights 3  very few nights 4  never 5  DON’T KNOW 9 |  |
| 204 | How likely is it that you or someone in your household will purchase a mosquito net in the next year – very likely, somewhat likely, or very unlikely? | very likely 1  somewhat likely 2  very unlikely 3  Don’t know 9 |  |
|  |  |  |  |
| 205 | Have you ever used a mosquito net for any of the following:  **MULTIPLE RESPONSES POSSIBLE**  **CIRCLE YES or NO for ALL RESPONSES**  **PROBE ONCE:** Anything else? | FISHING……………………………………………….Yes 1 No 2  COVERING/PROTECTION Yes 1 No2  SCREENS FOR WINDOWS Yes 1 No 2  CLOTHING Yes 1 No 2  OTHER Yes 1 No 2  (SPECIFY)  DON’T KNOW 9 |  |
| 206 | During this pregnancy, did you take any medicine to prevent you from getting malaria?  **EMPHASIZE ‘PREVENT’. DO NOT CIRCLE 1 IF SHE WAS ONLY GIVEN DRUGS BECAUSE SHE HAD MALARIA.** | yes 1  No 2  Don’t know 9 |  |

**SECTION 3: THREAT OF MALARIA**

| **PERCEIVED SUSCEPTIBILITY TO MALARIA:** I am going to read a series of statements about malaria to you and I would like you to tell me how much you agree with them.  For each statement, please tell me if you strongly agree, somewhat agree, somewhat disagree, or strongly disagree with it. INTERVIEWER: DO NOT READ DON’T KNOW OR UNCERTAIN RESPONSE AND ONLY USE OF RESPONDENT IS NOT ABLE TO PROVIDE ANOTHER ANSWER | | | | | | |
| --- | --- | --- | --- | --- | --- | --- |
|  |  | Strongly agree | somewhat agree | somewhat disagree | strongly disagree |  |
| 301 | During the rainy season, I worry almost every day that someone in my family will get malaria | 1 | 2 | 3 | 4 |  |
| 302 | When someone I know gets malaria, I usually expect them to completely recover in a few days | 1 | 2 | 3 | 4 |  |
| 303 | My children are so healthy that they would be able to recover from a case of malaria | 1 | 2 | 3 | 4 |  |
| 304 | People in this community only get malaria during rainy season | 1 | 2 | 3 | 4 |  |
| 305 | People only get malaria when there are lots of mosquitos | 1 | 2 | 3 | 4 |  |
| 306 | Pregnant women are still at risk for malaria even if they take the medicine that is meant to keep them from getting malaria | 1 | 2 | 3 | 4 |  |

| **PERCEIVED SEVERITY OF MALARIA:** I am going to read a series of statements to you and I would like you to tell me how much you agree with them.  For each statement, please tell me if you strongly agree, somewhat agree, somewhat disagree, or strongly disagree with it. INTERVIEWER: DO NOT READ DON’T KNOW OR UNCERTAIN RESPONSE AND ONLY USE OF RESPONDENT IS NOT ABLE TO PROVIDE ANOTHER ANSWER | | | | | |
| --- | --- | --- | --- | --- | --- |
|  |  | Strongly agree | somewhat agree | somewhat disagree | strongly disagree |
| 401 | I don’t worry about malaria because it can be easily treated | 1 | 2 | 3 | 4 |
| 402 | Every case of malaria can potentially lead to death | 1 | 2 | 3 | 4 |
| 403 | I cannot remember the last time someone I know became dangerously sick with malaria | 1 | 2 | 3 | 4 |
| 404 | Nearly every year, someone in this community gets a serious case of malaria | 1 | 2 | 3 | 4 |
|  |  |  |  |  |  |

**SECTION 5: SELF-EFFICACY QUESTIONS**

| I am going to ask you about a series of actions you could take, and I would like you to tell me how confident you are that you could actually do that action successfully.  For each action, please tell me if you think you definitely could, probably could, probably could not or definitely could not do each action successfully. INTERVIEWER: DO NOT READ DON’T KNOW OR UNCERTAIN RESPONSE AND ONLY USE IF RESPONDENT IS NOT ABLE TO PROVIDE ANOTHER ANSWER | | | | | |
| --- | --- | --- | --- | --- | --- |
|  |  | DEFINITELY COULD | PROBABLY COULD | PROBABLY COULD NOT | DEFINITELY COULD NOT |
| 501 | I can easily protect myself from getting malaria | 1 | 2 | 3 | 4 |
| 502 | I am confident that I can get enough bed nets to cover all of the sleeping spaces in my household | 1 | 2 | 3 | 4 |
| 503 | I am confident that I can sleep under a bed net for the entire night when there are lots of mosquitoes | 1 | 2 | 3 | 4 |
| 504 | I am confident that I can sleep under a bed net for the entire night when there are few mosquitos | 1 | 2 | 3 | 4 |
| 505 | I am confident that I can consistently use a bed net in rooms that are also used during the day for eating or sitting | 1 | 2 | 3 | 4 |

**SECTION 6: RESPONSE EFFICACY QUESTIONS**

|  |  | Strongly agree | somewhat agree | somewhat disagree | strongly disagree |
| --- | --- | --- | --- | --- | --- |
| 601 | My chances of getting malaria are same whether or not I sleep under a bed net | 1 | 2 | 3 | 4 |
| 602 | Many people who sleep under mosquito nets still get malaria | 1 | 2 | 3 | 4 |
| 603 | Bed nets only prevent mosquito bites when used with certain types of beds | 1 | 2 | 3 | 4 |
| 604 | I have noticed my family gets less sick often since we began sleeping under nets. | 1 | 2 | 3 | 4 |
| 605 | More expensive bed nets are more effective than less expensive or free bed nets | 1 | 2 | 3 | 4 |
| 606 | The insecticide on bed nets can be dangerous to people who sleep under them | 1 | 2 | 3 | 4 |

**SECTION 7: EXPOSURE TO MALARIA MESSAGES**

| NO. | QUESTION | CODING CATEGORIES | SKIP |
| --- | --- | --- | --- |
| 701 | How often do you listen to the radio in a week? | None 0  Once a week 1  2-3 times a week 2  4-5 time a week 3  More than 5 times a week 4 |  |
| 702 | How many times in a week do you watch television? | None 0  Once a week 1  2-3 times a week 2  4-5 time a week 3  More than 5 times a week 4 |  |
| 703 | In the past year have you heard or seen any messages about malaria prevention or treatment? | Yes 1  No 2  Don’t know 9 |  |
| 704 | What were these messages about?  PROBE ONCE: Anything else? ALLOW MULTIPLE RESPONSES | ITNs/Bednets 1  Retreatment 2  Act 3  Treatment For Pregnant Women 4  TREATMENT FOR SMALL CHILDREN  Severity Of Malaria 5  Risk Of Malaria 6  Indoor Residual Spraying 7  Other 8  Don’t know 9 |  |

NFT3PWXH
