# Supplementary material for: The role of perceived threat and self-efficacy in the use of Insecticide Treated Bednets (ITNs) to prevent malaria among pregnant women in Tororo District, Uganda
Source: PLoS One. 2023 Jul 26;18(7):e0289097. doi: 10.1371/journal.pone.0289097 (PMC10370871; doi:10.1371/journal.pone.0289097)
Supplement: Attachment — Submitted filename: Final Response to Reviewer comments_10_7_22.docx [file pone.0289097.s005.docx]

Dear Editor,

We thank the reviewers for their careful and attentive comments on our manuscript. Below, we respond point-by-point to the comments and suggestions and demonstrate changes made to the paper.

Sincere thanks,

When submitting your revision, we need you to address these additional requirements.

1. Please ensure that your manuscript meets PLOS ONE's style requirements, including those for file naming. The PLOS ONE style templates can be found at 
<https://journals.plos.org/plosone/s/file?id=wjVg/PLOSOne_formatting_sample_main_body.pdf> and 
<https://journals.plos.org/plosone/s/file?id=ba62/PLOSOne_formatting_sample_title_authors_affiliations.pdf>

2. You indicated that you had ethical approval for your study. In your Methods section, please ensure you have also stated whether you obtained consent from parents or guardians of the minors included in the study or whether the research ethics committee or IRB specifically waived the need for their consent.

*Consent was received individually from each of the study participants. According to the Uganda IRB Ethical guidelines section* ***5.8. Informed Consent by Mature and Emancipated Minor;*** *Emancipated minors (14-17) are individuals below the age of majority (****(****14-17) who are pregnant, married or have a child or cater for their own livelihood are considered emancipated minors. Mature and emancipated minors may independently provide informed consent to participate in research.*

3. Thank you for stating the following financial disclosure: 
   "NO - The funders had no role in study design, data collection and analysis, decision to publish, or preparation of the manuscript."

At this time, please address the following queries:

a) Please clarify the sources of funding (financial or material support) for your study. List the grants or organizations that supported your study, including funding received from your institution.

*This has been updated. This study received a partial financial contribution (approx.$ 2,100) from the Malaria consortium Uganda.*

b) State what role the funders took in the study. If the funders had no role in your study, please state: “The funders had no role in study design, data collection and analysis, decision to publish, or preparation of the manuscript.”

*The funders had no role in the study design, data collection, analysis decision to publish or preparation of the manuscript*

c) If any authors received a salary from any of your funders, please state which authors and which funders.
d) If you did not receive any funding for this study, please state: “The authors received no specific funding for this work.”

*“The authors received no specific funding for this work.”*

Please include your amended statements within your cover letter; we will change the online submission form on your behalf.

*This has been done*

4.Thank you for stating the following in the Acknowledgments Section of your manuscript: 
   "The authors wish to thank the Tororo district general hospital authorities for their support in the data collection and operationalisation of the study and the respondents of the survey questionnaires. This work was partly funded by the Malaria Consortium Uganda. The contents do not necessarily reflect the views of Malaria Consortium Uganda. "

We note that you have provided funding information that is not currently declared in your Funding Statement. However, funding information should not appear in the Acknowledgments section or other areas of your manuscript. We will only publish funding information present in the Funding Statement section of the online submission form. 
Please remove any funding-related text from the manuscript and let us know how you would like to update your Funding Statement. Currently, your Funding Statement reads as follows: 
  "NO - The funders had no role in study design, data collection and analysis, decision to publish, or preparation of the manuscript."

Please include your amended statements within your cover letter; we will change the online submission form on your behalf.

*This has been amended to read; While the study received a partial financial contribution from the Malaria consortium Uganda, the funders had no role in the study design, data collection, analysis decision to publish or preparation of the manuscript.*

5. Your ethics statement should only appear in the Methods section of your manuscript. If your ethics statement is written in any section besides the Methods, please move it to the Methods section and delete it from any other section. Please ensure that your ethics statement is included in your manuscript, as the ethics statement entered into the online submission form will not be published alongside your manuscript.

6. We note you have included a table to which you do not refer in the text of your manuscript. Please ensure that you refer to Table 2,4 and 5 in your text; if accepted, production will need this reference to link the reader to the Table.

*The table numbers have been updated and the text now refers to all the tables*

7. Please include captions for your Supporting Information files at the end of your manuscript, and update any in-text citations to match accordingly. Please see our Supporting Information guidelines for more information: <http://journals.plos.org/plosone/s/supporting-information>.

*Submitted the dataset under Supporting Information files. The file is labeled S1_Dataset.*

Reviewer #1: 

Reviewer comments
General comments: This is an important and unique study on ITN use among pregnant women looking the important role played by perceived susceptibility, severity, self-efficacy and response efficacy. However, this paper needs some major revisions before being considered for publication.

*Thank you for the comments. Revision have been made in the paper accordingly*

Specific comments
Introduction: “While the Extended Parallel Process Model has been tested in several interventions including workplace safety [13], asthma intervention programs [14], and physicians’ decision to test patients for kidney disease [15] among others, there is no known study that has tested the application of the model in a malaria setting, specifically ITN use among pregnant women”.

#1 This is a very important statement as it highlights the contribution this study will add to the body of knowledge. Could the authors add to this statement by highlighting the importance of the four EPPM construct in malaria prevention (i.e ITN use) and how this had impacted other health interventions and behaviours

The following statement has been added; *For interventions to be effective, communication messages should be aimed at increasing risk perceptions related to the threat of malaria, specifically perceptions that the disease can have severe outcomes and that everyone is susceptible, with pregnant women and children under the age of five being particularly vulnerable. This should be accompanied with appropriate efficacy messages to ensure danger control, including self-efficacy to use ITNs and promoting perceptions that ITNs are effective in controlling the transmission of malaria. Combining the four EPPM constructs is theoretically a recipe for behavioral change.*

*Also added; In their study, Roberto et. al confirmed their prediction that physicians who perceived kidney disease to be a greater threat to patients and had higher efficacy, demonstrated greater intentions and behavior to test their patients' level of kidney functioning.*

Methods-Questionnaire
#2 I am wondering why the authors measured the four EPPM construct using 4-point scale response instead of the known 5-point likert scale response.

*We thank the reviewer for their observation. While a five-point Likert scale is more commonly utilized, four-point scales are frequently used. The justification for using a four-point scale is that they are easier to use especially where literacy levels are low.*

#3 Authors should give details on how the 4-point scale responses were recategorized into high, moderate and low

*The responses to the items were summed and then tertiles of the score were used to create high, moderate and low categories.*

#4 I am wondering why the authors considered only age, marital status, educational attainment and socio-economic status as the covariates. Were they guided by literature?
I thought a decision of a pregnant woman to sleep under ITN is also highly influenced by their partners (husband) characteristics. It could be interesting to control for the effect of the husband characteristics on EPPM construct and pregnant women ITN use relationship since the study setting (Uganda) is where African’s men are major decision makers.

*The reviewer makes an excellent point about the gendered nature of household decision-making. Unfortunately, we did not measure this and therefore cannot control for it in the analysis. In conceptualizing the study, we aimed at testing the four variables of the EPPM and the commonly used determinants for net use. The study did not measure any partner characteristics.*

#5 Can the authors kindly provide details on how “consistency of ITN use” was measured?
What constitutes consistency?

*Consistency of ITN use was measured using an item that asked; “In general, how often do you sleep under a mosquito bed net? with response categories “every night, most nights, some nights, very few nights, never”. Respondents who answered ““Every night” were categorized as consistent ITN users and all other responses were recoded as “inconsistent”.*

Ethics
“Ethical approval was obtained from the University of Witwatersrand Faculty of Health
Sciences Human Research Ethics Committee, in South Africa, the School of Health Sciences
Research and Ethics committee at Makerere University in Uganda and the Uganda National
Council for Science and Technology”.

#5 Could authors kindly provided the ethical protocol number?

*The University of Witwatersrand Faculty of Health Sciences Human Research Ethics Committee protocol number is: M140860 and Uganda National Council for Science and Technology research registration number is: SS3656*

Results
\# 6 In Table 4 and 5. I am wondering whether the authors used row or column percentage in calculating the association between socio-demographic characteristics and ITN use. This is because I am struggling to see how the percentage were obtained. Authors should kindly clarify

*In Table 4 (now Table 3) we used row percentages. In Table 5 (now table 4) we used column percentages.*

\# 7 In Table 5, Authors considered “inconsistent ITN use and no ITN use as one” Please clarify or provide some explanation to why a pregnant woman who is inconsistently using ITN will be considered as the same with another who is not using ITN.
*The table heading has been updated as the column should have stated no ITN use the previous night.*

Conclusion
“We found that the threat and self-efficacy constructs in the model explained consistent ITN use among pregnant women. These findings can guide future interventions to maintain ITN use in malaria endemic settings”.
#8. Authors should give a detailed explanation on how EPPM constructs can guide future ITN usage in malaria endemic areas.

*The following statement has been added; Future communication interventions need to utilize messages and other activities to increase self-efficacy to use ITNs together with sustaining high perceptions of the threat of malaria in order to improve Net use.*

Reviewer #2: Thank editor and authors for giving me a chance to read this manuscript. The study pointed out a public health problem i.e., ITN use among pregnant women. Overall, the manuscript is well written but for me, I think the methods and discussion need major attention to publish it at the PlosOne.

*These have been updated*

Reviewer 2 Comments;

- Use standard keywords (e.g., mesh-engine)

*The keywords have been modified to align better with MeSH.*

- Word usage should be consistent (e.g., AOR or aOR?)

*This has been corrected to AOR.*

- What does it mean the communication interventions? Use the specific word.

*This term is used to refer to communications aimed at the promotion of utilization of ITN*

**Introduction**

- Expand/ Include absolute number for magnitude of malaria problem in Uganda.

- WHO: only use long form in first appearance

- Needs description about malaria problem among pregnant women. How were previous study findings?

**Methods**

- Needs detailed inclusion and exclusion criteria for sampling the pregnant women;

*All pregnant women attending ANC aged 14 and older were eligible to participate in the study.*

- Includes detailed sample size calculation

*Using the STATA command – Sampsi, the sample size was calculated based on an assumption that 35 percent of women with low perceived severity and efficacy would be ITN users and 55% among those with high perceived severity and high efficacy would be ITN users. The 35% was a conservative estimate based on the nationwide figures for ITN use among pregnant women in 2009 which was estimated at 44% (52). This sample size was calculated assuming a power of 0.80 and an alpha of 0.05. A sample size of 212 women was recommended for the study. This was increased by approximately 15% to account for non-response or refusals yielding a target sample of 245.*

- Given that there were a lot of IRB committees there, put IRB-number of each approval.

*This has been included as per the responses to Review #1 comments.*

- Are there any criteria for independent variables to be included in logistics regression models?

*All models controlled for education attainment and Socio-Economic Status which were considered to be potential confounders.*

- Consisting of a map locating study area shall be considered for the readers know the geographical situation.

*The map showing the study area has been added*

**Results**

- Why did the samples different for educational attainment? How did you control it during analysis?

*28 participants did not answer the question about educational attainment. We did not impute for missing data. This means that our sample size for the regression was slightly reduced.*

- How did you get sample size 46 for asking reasons for non-ITN use?

*46 participants reported not using ITN the previous night. Only those were asked for reasons for non-use.*

- Usually, we didn’t use the word Associations for the results out from Chi-square only. (Your table 4 and 5)

*This reference to association has been changed to “Relation” where chi-square was used.*

- Table 5: How did you categorize Low, moderate, and high groups for the independent variables? Are there any theories or references?

*Same response as above to reviewer 1 comment on the categorization.*

- For logistics regression, better to include (n) for each group rather than putting only AOR and 95%CIs.

*The (n) has been added to the title.*

**Discussion**

- Not only referencing to other studies, but also points of view from authors should be added based on contexts of study areas on why these findings come out?

- Some sentences are misleading (e.g., Page 18, Line 306-308 I believed current study did not research on insecticide resistance).

*The statement on Insecticide resistance is in reference to another study which linked increasing resistance of anopheles mosquitoes to the pyrethrum insecticides – the substance used to treat nets – hence potentially making them seamless effective. Response efficacy is based on a belief that the proposed intervention works, such as ITNs for prevention of malaria. Anything that decreases the perception around net effectiveness may impact on response efficacy.*

**Other comments**

- Language should be improved throughout the manuscript.

*This has been addressed*

- Although the study was conducted in 2015, better to use the data from recent reports or papers. Almost all references are a bit old and need to be changed with recent ones.

- Also, references styles need to be rechecked and revised. (e.g., journal names should follow PubMed acronyms). Check against Plos in-house style.

- Include study tools as supplementary file(s).

*This has been added as S2.*
