# Supplementary material for: The role of perceived threat and self-efficacy in the use of Insecticide Treated Bednets (ITNs) to prevent malaria among pregnant women in Tororo District, Uganda
Source: PLoS One. 2023 Jul 26;18(7):e0289097. doi: 10.1371/journal.pone.0289097 (PMC10370871; doi:10.1371/journal.pone.0289097)
Supplement: Attachment — Submitted filename: Reviewer comment_R2.docx [file pone.0289097.s006.docx]

**Abstract**

- Use standard keywords (e.g., mesh-engine) (I did not see any revision)

- Word usage should be consistent (e.g., AOR or aOR?)

- What does it mean the communication interventions? Use the specific word. (Thanks for the explanation but it is not a scientific word, and I believed the meaning will be misleading for some readers)

**Introduction**

- Expand/ Include absolute number for magnitude of malaria problem in Uganda. (Not only mentioned the malaria problem is high, please add the real number of malaria problem e.g we can reference to WHO world malaria report 2022)

- WHO: only use long form in first appearance

- Needs description about malaria problem among pregnant women. How were previous study findings? (I did not see author response on it)

**Methods**

- Needs detailed inclusion and exclusion criteria for sampling the pregnant women (how was the exclusion criteria for example pregnant women migrated from another area)

- Includes detailed sample size calculation

- Given that there were a lot of IRB committees there, put IRB-number of each approval.

- Are there any criteria for independent variables to be included in logistics regression models?

- Consisting of a map locating study area shall be considered for the readers know the geographical situation.

**Results**

- Why did the samples different for educational attainment? How did you control it during analysis?

- How did you get sample size 46 for asking reasons for non-ITN use?

- Usually, we didn’t use the word Associations for the results out from Chi-square only. (Your table 4 and 5) (should correct in the paragraph as well)

- Table 5: How did you categorize Low, moderate, and high groups for the independent variables? Are there any theories or references? (Please also add a relevant citation for the criteria use e.g., bloom classification)

- For logistics regression, better to include (n) for each group rather than putting only AOR and 95%CIs.

**Discussion**

- Not only referencing to other studies, but also points of view from authors should be added based on contexts of study areas on why these findings come out?

- Some sentences are misleading (e.g., Page 18, Line 306-308 I believed current study did not research on insecticide resistance). (The literatures might be relevant with the title, but we usually discussed what we found from the study findings/ results. I still believed current study did not research on insecticide resistance)

**Other comments**

- Language should be improved throughout the manuscript. (I still see many errors throughout the manuscript)

- Although the study was conducted in 2015, better to use the data from recent reports or papers. Almost all references are a bit old and need to be changed with recent ones.

- Also, references styles need to be rechecked and revised. (e.g., journal names should follow PubMed acronyms). Check against Plos in-house style. (I did not see author corrections on the reference styles. Most of the reference are wrong especially authors names, year of publication and the journal names)

- Include study tools as supplementary file(s).
