# Supplementary material for: The role of perceived threat and self-efficacy in the use of Insecticide Treated Bednets (ITNs) to prevent malaria among pregnant women in Tororo District, Uganda
Source: PLoS One. 2023 Jul 26;18(7):e0289097. doi: 10.1371/journal.pone.0289097 (PMC10370871; doi:10.1371/journal.pone.0289097)
Supplement: Attachment — Submitted filename: Revised manuscript Rebuttal to comments.docx [file pone.0289097.s007.docx]

Dear Editor,

We thank the reviewers for their careful and attentive comments on our manuscript. Below, we respond point-by-point to the comments and suggestions and demonstrate changes made to the paper. We acknowledge that some of the comments had not been fully addressed in the previous round of revisions. We hope that we have now done justice to the quality comments

Sincere thanks,

When submitting your revision, we need you to address these additional requirements.

Abstract

- Use standard keywords (e.g., mesh-engine) (I did not see any revision)

*Thank you for flagging the need to ensure that keywords match mesh-engine. This has been updated to the standard keywords e.g. insecticide treated bed nets, risk reduction behaviors.*

- Word usage should be consistent (e.g., AOR or aOR?)

*This has been updated*

- What does it mean the communication interventions? Use the specific word.

*Thank you for pointing out the need to clarify the term used. This has been updated to social and behavior change communication interventions*

Introduction

- Expand/ Include absolute number for magnitude of malaria problem in Uganda. (Not only mentioned the malaria problem is high, please add the real number of malaria problem e.g we can reference to WHO world malaria report 2022)

*This paragraph has been updated to include numbers; incidence rates at above 154 cases per 1,000 people*

- WHO: only use long form in first appearance.

*This has been updated.*

- Needs description about malaria problem among pregnant women. How were previous study findings? (I did not see author response on it)

*Thanks for pointing out the gap. We added the following: Several poor health outcomes are associated with malaria in pregnancy including stillbirth, preterm birth, maternal and neonatal mortality, congenital malaria, maternal anaemia and low birth weight [4] A study by Namusoke and others noted that malaria infection rates among pregnant women were 15.5% (59/380) of active infections and 4.5% (17/380) of past infections [5].*

Methods

- Needs detailed inclusion and exclusion criteria for sampling the pregnant women (how was the exclusion criteria for example pregnant women migrated from another area)

*Thank you for the comment. We have added sentence.*

- Includes detailed sample size calculation

*A detailed description of sample size calculation has been added*

- Given that there were a lot of IRB committees there, put IRB-number of each approval.

*This has been updated, with the respective IRB numbers included as follows; University of Witwatersrand Faculty of Health Sciences Human Research Ethics Committee, in South Africa (#M140860), the School of Health Sciences Research and Ethics committee at Makerere University in Uganda (#256) and the Uganda National Council for Science and Technology (# SS 3656).*

- Are there any criteria for independent variables to be included in logistics regression models?

*We selected variables that were statistically significant or approaching significance*

- Consisting of a map locating study area shall be considered for the readers know the

geographical situation.

*This has been updated and the map included as Figure 2*

Results

- Why did the samples different for educational attainment? How did you control it during analysis?

*Educational attainment data was missing for 28 participants. We controlled for educational attainment in the analysis. But we didn’t impute for missing data.*

- How did you get sample size 46 for asking reasons for non-ITN use?

*Of the 49 participants who reported not using an ITN the previous night, 46 provided a reason. We added a sentence to explain this in the results.*

- Usually, we didn’t use the word Associations for the results out from Chi-square only. (Your table 4 and 5) (should correct in the paragraph as well)

*Thank you for pointing this out. We have made changes to the paragraphs as well.*

- Table 5: How did you categorize Low, moderate, and high groups for the independent

variables? Are there any theories or references? (Please also add a relevant citation for the criteria use e.g., bloom classification)

*We used tertiles to categorise a continuous variable into the three groups; Low moderate and high.*

- For logistics regression, better to include (n) for each group rather than putting only AOR and 95% CIs.

*The “n” is included in the table title*

Discussion

- Some sentences are misleading (e.g., Page 18, Line 306-308 I believed current study did not research on insecticide resistance). (The literatures might be relevant with the title, but we usually discussed what we found from the study findings/ results. I still believed current study did not research on insecticide resistance)

*We removed the sentences on insecticide resistance as we agree that the text is better without it.*

Other comments

- Language should be improved throughout the manuscript. (I still see many errors throughout the manuscript)

*This has been updated.*

- Although the study was conducted in 2015, better to use the data from recent reports or papers. Almost all references are a bit old and need to be changed with recent ones.

*We have added some more recent references.*

- Also, references styles need to be rechecked and revised. (e.g., journal names should follow PubMed acronyms). Check against Plos in-house style. (I did not see author corrections on the reference styles. Most of the reference are wrong especially authors names, year of publication and the journal names)

*We have checked and edited the references*

- Include study tools as supplementary file(s).

*This has been added as S2.*
