# Supplementary material for: The role of perceived threat and self-efficacy in the use of Insecticide Treated Bednets (ITNs) to prevent malaria among pregnant women in Tororo District, Uganda
Source: PLoS One. 2023 Jul 26;18(7):e0289097. doi: 10.1371/journal.pone.0289097 (PMC10370871; doi:10.1371/journal.pone.0289097)
Supplement: Attachment — Submitted filename: Reviewer comment.docx [file pone.0289097.s004.docx]

**Abstract**

- Use standard keywords (e.g., mesh-engine)

- Word usage should be consistent (e.g., AOR or aOR?)

- What does it mean the communication interventions? Use the specific word.

**Introduction**

- Expand/ Include absolute number for magnitude of malaria problem in Uganda.

- WHO: only use long form in first appearance

- Needs description about malaria problem among pregnant women. How were previous study findings?

**Methods**

- Needs detailed inclusion and exclusion criteria for sampling the pregnant women

- Includes detailed sample size calculation

- Given that there were a lot of IRB committees there, put IRB-number of each approval.

- Are there any criteria for independent variables to be included in logistics regression models?

- Consisting of a map locating study area shall be considered for the readers know the geographical situation.

**Results**

- Why did the samples different for educational attainment? How did you control it during analysis?

- How did you get sample size 46 for asking reasons for non-ITN use?

- Usually, we didn’t use the word Associations for the results out from Chi-square only. (Your table 4 and 5)

- Table 5: How did you categorize Low, moderate, and high groups for the independent variables? Are there any theories or references?

- For logistics regression, better to include (n) for each group rather than putting only AOR and 95%CIs.

**Discussion**

- Not only referencing to other studies, but also points of view from authors should be added based on contexts of study areas on why these findings come out?

- Some sentences are misleading (e.g., Page 18, Line 306-308 I believed current study did not research on insecticide resistance).

**Other comments**

- Language should be improved throughout the manuscript.

- Although the study was conducted in 2015, better to use the data from recent reports or papers. Almost all references are a bit old and need to be changed with recent ones.

- Also, references styles need to be rechecked and revised. (e.g., journal names should follow PubMed acronyms). Check against Plos in-house style.

- Include study tools as supplementary file(s).
